# (2*E*,2′*E*)-1,1′-Bis(6-chloro-2-methyl-4-phenyl­quinolin-3-yl)-3,3′-(1,4-phenyl­ene)diprop-2-en-1-one ethyl acetate disolvate

**DOI:** 10.1107/S1600536812049422

**Published:** 2012-12-08

**Authors:** Allaoua Kedjadja, Rachid Merdes, Sofiane Bouacida, Thierry Roisnel, Ali Belfaitah

**Affiliations:** a Laboratoire de Chimie Appliquée, Faculté des Sciences, Université de Guelma 24000, Algeria; b Unité de Recherche de Chimie de l’Environnement et Moléculaire Structurale, CHEMS, Université Mentouri-Constantine, 25000 Algeria; c Centre de Difractométrie X, UMR 6226 CNRS Unité Sciences Chimiques de Rennes, Université de Rennes I, 263 Avenue du Général Leclerc, 35042 Rennes, France; d Laboratoire des Produits Naturels, d’Origine Végétale et de Synthèse Organique, PHYSYNOR, Université Mentouri-Constantine, 25000 Constantine, Algeria

## Abstract

In the title solvate, C_44_H_30_Cl_2_N_2_O_2_·2C_4_H_8_O_2_, the complete polycyclic mol­ecule is generated by inversion symmetry. The dihedral angle between the quinolyl ring system (*Q;* r.m.s. deviation = 0.020 Å) and the pendant phenyl ring is 78.80 (6)°; the dihedral angle between *Q* and the central benzene ring is 85.92 (7)°. In the crystal, the components are linked by C—H⋯O and C—H⋯π inter­actions, generating (110) layers. Weak aromatic π–π stacking [centroid–centroid distances = 3.7025 (11) and 3.8124 (10) Å] is also observed.

## Related literature

For our previous studies in the area of potentially bioactive mol­ecules, see: Menasra *et al.* (2005 ▶); Kedjadja *et al.* (2004 ▶). For further synthetic details, see: Wang *et al.* (2006 ▶).
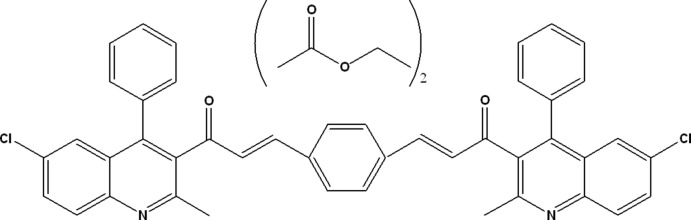


## Experimental

### 

#### Crystal data

C_44_H_30_Cl_2_N_2_O_2_·2C_4_H_8_O_2_
*M* *_r_* = 865.81
Triclinic, 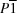

*a* = 9.9851 (3) Å
*b* = 10.0086 (2) Å
*c* = 11.3676 (3) Å
α = 102.350 (1)°
β = 97.108 (1)°
γ = 95.290 (2)°
*V* = 1092.94 (5) Å^3^
*Z* = 1
Mo *K*α radiation
μ = 0.20 mm^−1^
*T* = 150 K
0.25 × 0.15 × 0.1 mm

#### Data collection

Bruker APEXII diffractometer
Absorption correction: multi-scan (*SADABS*; Sheldrick, 2002 ▶) *T*_min_ = 0.884, *T*_max_ = 0.980
18119 measured reflections
4866 independent reflections
4035 reflections with *I* > 2σ(*I*)
*R*_int_ = 0.026

#### Refinement

*R*[*F* ^2^ > 2σ(*F* ^2^)] = 0.048
*wR*(*F* ^2^) = 0.140
*S* = 1.04
4866 reflections
283 parameters
H-atom parameters constrained
Δρ_max_ = 1.02 e Å^−3^
Δρ_min_ = −0.42 e Å^−3^

### 

Data collection: *APEX2* (Bruker, 2001 ▶); cell refinement: *SAINT* (Bruker, 2001 ▶); data reduction: *SAINT*; program(s) used to solve structure: *SIR2002* (Burla *et al.*, 2005 ▶); program(s) used to refine structure: *SHELXL97* (Sheldrick, 2008 ▶); molecular graphics: *ORTEP-3* (Farrugia, 2012 ▶) and *DIAMOND* (Brandenburg & Berndt, 2001 ▶); software used to prepare material for publication: *WinGX* (Farrugia, 2012 ▶).

## Supplementary Material

Crystal structure: contains datablock(s) global, I. DOI: 10.1107/S1600536812049422/hb7004sup1.cif

Structure factors: contains datablock(s) I. DOI: 10.1107/S1600536812049422/hb7004Isup2.hkl

Additional supplementary materials:  crystallographic information; 3D view; checkCIF report

## Figures and Tables

**Table 1:** Hydrogen-bond geometry (Å, °) *Cg*1 is the centroid of the C12–C17 ring.

*D*—H⋯*A*	*D*—H	H⋯*A*	*D*⋯*A*	*D*—H⋯*A*
C13—H13⋯O19^i^	0.95	2.54	3.218 (2)	128
C23—H23⋯O53	0.95	2.57	3.468 (2)	157
C24—H24⋯O56^ii^	0.95	2.48	3.410 (3)	165
C51—H51*B*⋯*Cg*1^iii^	0.98	2.76	3.627 (3)	147

Symmetry codes: (i) 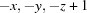; (ii) 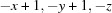; (iii) 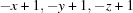.

**Fig. 1.**
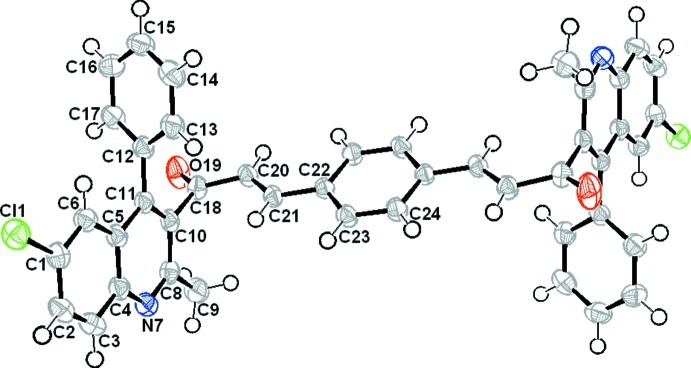
The molecular geometry of (I) with displacement ellipsoids drawn at the 50% probability level. Only the contents of the asymmetric unit are numbered. The two ethyl acetate co-crystalized molecules were omitted for clarity.

**Fig. 2.**
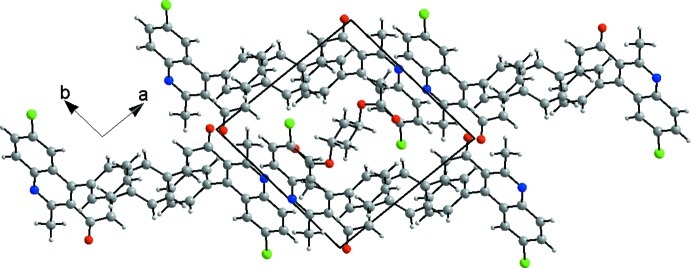
A diagram of the layered crystal packing of (I) viewed down the c axis.

## supplementary crystallographic information

### Comment

In continuation of our interest related to the synthesis and structures of potentially bioactive products (Kedjadja *et al.* 2004; Menasra *et al.* 2005), we report herein the synthesis and the structure determination of the title compound, (I). The reactivity of this compound and its analogues toward nucleophiles is under investigation.

The molecular geometry and the atom-numbering scheme of (I) are shown in Fig. 1. The asymmetric unit of (I)consists of one-half of the molecule, with the other half generated by a crystallographic inversion centre. In the title molecule the centrosymmetric phenyl ring is attached to two prop-2-en-1-one linked to two 6-chloro-2-methyl-4-phenylquinolin-3-yl and two molecules of ethyl acetate are co-crystalized with it. The two rings of quinolyl group are fused in axial fashion and form adihedral angle of 1.72 (5)° and this quasi plane system forms a dihedral angle of 78.80 (6)° with the phenyl ring (C12—C17) attached to quinolyl moiety. The crystal packing can be described as layers in zigzag parallel to the (110) plane. (Fig. 2). It features C—H···O and C—H···π interactions (Table. 1) and strong π-π stacking interactions between quinolyl rings with a centroid-centroid distance of 3.7025 (11) and 3.8124 (10)å. These interactions link the molecules within the layers and also link the layers together, reinforcing the cohesion of the structure.

### Experimental

A mixture of 2-aminobenzophénone (1.0 mmol), acetylacetone (1.2 mmol), water (1 ml) and 1.0 eq. of 1 N HCl, gave the corresponding 1-(2-methyl-4-phenylquinolin-3-yl) ethanone as a white solid in 86% yield, according to the procedure reported by Wang *et al.* (2006). Next, the title compound was prepared in 75% of yield, by an aldol condensation reaction of the Friedländer product with 0.5 eq. of terephthalaldehyde in an ethanolic solution of NaOH at room temperature. Colourless prisms of (I) were obtained by crystallization from ethyl acetate/petroleum ether (1/1) solution.

### Refinement

Approximate positions for all the H atoms were first obtained from the difference electron density map. However, the H atoms were situated into idealized positions and the H-atoms have been refined within the riding atom approximation. The applied constraints were as follow: C_aryl_—H_aryl_ = 0.95 Å; C_methylene_—H_methylene_ = 0.99 Å and C_methyl_—H_methyl_ = 0.98 Å and; The idealized methyl group was allowed to rotate about the C—C bond during the refinement by application of the command AFIX 137 in *SHELXL97* (Sheldrick, 2008). *U*_iso_(H_methyl_) = 1.5*U*_eq_(C_methyl_) or *U*_iso_(H_aryl_ or H_methylene_) = 1.2 *U*_eq_(C_aryl_ or C_methylene_).

### Crystal data

**Table:**

C_44_H_30_Cl_2_N_2_O_2_·2C_4_H_8_O_2_	*Z* = 1
*M**_r_* = 865.81	*F*(000) = 454
Triclinic, *P*1	*D*_x_ = 1.315 Mg m^−^^3^
Hall symbol: -P 1	Mo *K*α radiation, λ = 0.71073 Å
*a* = 9.9851 (3) Å	Cell parameters from 8289 reflections
*b* = 10.0086 (2) Å	θ = 2.5–27.5°
*c* = 11.3676 (3) Å	µ = 0.20 mm^−^^1^
α = 102.350 (1)°	*T* = 150 K
β = 97.108 (1)°	Prism, colourless
γ = 95.290 (2)°	0.25 × 0.15 × 0.1 mm
*V* = 1092.94 (5) Å^3^	

### Data collection

**Table:**

Bruker APEXII diffractometer	4035 reflections with *I* > 2σ(*I*)
Graphite monochromator	*R*_int_ = 0.026
CCD rotation images, thin slices scans	θ_max_ = 27.5°, θ_min_ = 2.8°
Absorption correction: multi-scan (*SADABS*; Sheldrick, 2002)	*h* = −12→12
*T*_min_ = 0.884, *T*_max_ = 0.980	*k* = −12→12
18119 measured reflections	*l* = −14→14
4866 independent reflections	

### Refinement

**Table:**

Refinement on *F*^2^	Primary atom site location: structure-invariant direct methods
Least-squares matrix: full	Secondary atom site location: difference Fourier map
*R*[*F*^2^ > 2σ(*F*^2^)] = 0.048	Hydrogen site location: inferred from neighbouring sites
*wR*(*F*^2^) = 0.140	H-atom parameters constrained
*S* = 1.04	*w* = 1/[σ^2^(*F*_o_^2^) + (0.0694*P*)^2^ + 0.8188*P*] where *P* = (*F*_o_^2^ + 2*F*_c_^2^)/3
4866 reflections	(Δ/σ)_max_ = 0.011
283 parameters	Δρ_max_ = 1.02 e Å^−^^3^
0 restraints	Δρ_min_ = −0.42 e Å^−^^3^

### Special details

**Table:**

Geometry. All e.s.d.'s (except the e.s.d. in the dihedral angle between two l.s. planes) are estimated using the full covariance matrix. The cell e.s.d.'s are taken into account individually in the estimation of e.s.d.'s in distances, angles and torsion angles; correlations between e.s.d.'s in cell parameters are only used when they are defined by crystal symmetry. An approximate (isotropic) treatment of cell e.s.d.'s is used for estimating e.s.d.'s involving l.s. planes.
Refinement. Refinement of *F*^2^ against ALL reflections. The weighted *R*-factor *wR* and goodness of fit *S* are based on *F*^2^, conventional *R*-factors *R* are based on *F*, with *F* set to zero for negative *F*^2^. The threshold expression of *F*^2^ > σ(*F*^2^) is used only for calculating *R*-factors(gt) *etc*. and is not relevant to the choice of reflections for refinement. *R*-factors based on *F*^2^ are statistically about twice as large as those based on *F*, and *R*- factors based on ALL data will be even larger.

### Fractional atomic coordinates and isotropic or equivalent isotropic displacement parameters (Å^2^)

**Table:**

	*x*	*y*	*z*	*U*_iso_*/*U*_eq_	
O53	0.31158 (16)	0.42524 (16)	0.11802 (16)	0.0417 (4)	
Cl1	0.31876 (5)	0.75570 (5)	0.69990 (4)	0.02960 (15)	
C1	0.23208 (18)	0.65060 (19)	0.56441 (16)	0.0234 (4)	
C2	0.15254 (19)	0.70928 (19)	0.48229 (18)	0.0265 (4)	
H2	0.1473	0.8057	0.4995	0.032*	
C3	0.08296 (19)	0.6252 (2)	0.37739 (18)	0.0265 (4)	
H3	0.0294	0.6642	0.3215	0.032*	
C4	0.08921 (18)	0.48125 (18)	0.35046 (16)	0.0220 (4)	
C5	0.17208 (17)	0.42485 (18)	0.43299 (16)	0.0209 (4)	
C6	0.24378 (18)	0.51286 (18)	0.54149 (16)	0.0226 (4)	
H6	0.2995	0.4763	0.5978	0.027*	
N7	0.01376 (16)	0.40156 (16)	0.24584 (14)	0.0248 (3)	
C8	0.01928 (18)	0.26750 (19)	0.22137 (16)	0.0232 (4)	
C9	−0.0649 (2)	0.1817 (2)	0.10685 (18)	0.0309 (4)	
H9A	−0.1169	0.2411	0.0654	0.046*	
H9B	−0.1277	0.112	0.1274	0.046*	
H9C	−0.0051	0.1361	0.0531	0.046*	
C10	0.10213 (17)	0.20250 (18)	0.29886 (16)	0.0210 (4)	
C11	0.17907 (17)	0.28022 (18)	0.40423 (16)	0.0206 (4)	
C12	0.26671 (18)	0.21649 (18)	0.48762 (16)	0.0214 (4)	
C13	0.20937 (19)	0.14663 (19)	0.56647 (18)	0.0262 (4)	
H13	0.1138	0.1385	0.5664	0.031*	
C14	0.2911 (2)	0.0889 (2)	0.64504 (18)	0.0300 (4)	
H14	0.2515	0.0424	0.6993	0.036*	
C15	0.4301 (2)	0.0989 (2)	0.64447 (18)	0.0305 (4)	
H15	0.4858	0.0584	0.6976	0.037*	
C16	0.4880 (2)	0.1681 (2)	0.56644 (19)	0.0303 (4)	
H16	0.5834	0.1745	0.5658	0.036*	
C17	0.40700 (19)	0.2279 (2)	0.48915 (17)	0.0265 (4)	
H17	0.4474	0.2769	0.437	0.032*	
C18	0.10551 (18)	0.04827 (18)	0.26507 (16)	0.0228 (4)	
O19	0.02241 (15)	−0.02933 (15)	0.29611 (15)	0.0380 (4)	
C20	0.21001 (18)	−0.00520 (18)	0.19329 (16)	0.0218 (4)	
H20	0.2189	−0.1008	0.1793	0.026*	
C21	0.29293 (17)	0.07597 (18)	0.14703 (15)	0.0202 (3)	
H21	0.2831	0.1714	0.1651	0.024*	
C22	0.39718 (17)	0.03406 (17)	0.07162 (15)	0.0191 (3)	
C23	0.47009 (18)	0.13423 (18)	0.02708 (16)	0.0216 (4)	
H23	0.4496	0.2266	0.0455	0.026*	
C24	0.57142 (18)	0.10142 (18)	−0.04320 (16)	0.0218 (4)	
H24	0.6197	0.1711	−0.0722	0.026*	
C51	0.5390 (2)	0.5546 (3)	0.1748 (2)	0.0432 (5)	
H51A	0.5797	0.4728	0.139	0.065*	
H51B	0.6037	0.6128	0.2421	0.065*	
H51C	0.5165	0.6066	0.1127	0.065*	
C52	0.4113 (2)	0.5106 (2)	0.2220 (2)	0.0398 (5)	
H52A	0.4331	0.4563	0.2836	0.048*	
H52B	0.3716	0.5928	0.2611	0.048*	
C54	0.2331 (2)	0.4949 (3)	0.0581 (2)	0.0412 (5)	
C55	0.1442 (3)	0.3999 (3)	−0.0465 (2)	0.0465 (6)	
H55A	0.0557	0.376	−0.0225	0.07*	
H55B	0.1867	0.3159	−0.0712	0.07*	
H55C	0.1314	0.4451	−0.1148	0.07*	
O56	0.23575 (18)	0.61974 (16)	0.08844 (18)	0.0503 (5)	

### Atomic displacement parameters (Å^2^)

**Table:**

	*U*^11^	*U*^22^	*U*^33^	*U*^12^	*U*^13^	*U*^23^
O53	0.0426 (9)	0.0300 (8)	0.0574 (10)	0.0100 (7)	0.0142 (8)	0.0142 (7)
Cl1	0.0318 (3)	0.0239 (2)	0.0291 (2)	0.00118 (18)	0.00172 (18)	−0.00038 (18)
C1	0.0207 (9)	0.0231 (9)	0.0252 (9)	0.0010 (7)	0.0050 (7)	0.0024 (7)
C2	0.0250 (9)	0.0198 (8)	0.0357 (10)	0.0050 (7)	0.0068 (8)	0.0062 (8)
C3	0.0255 (9)	0.0245 (9)	0.0312 (10)	0.0074 (7)	0.0035 (7)	0.0087 (8)
C4	0.0207 (9)	0.0222 (9)	0.0243 (9)	0.0045 (7)	0.0069 (7)	0.0054 (7)
C5	0.0192 (8)	0.0214 (8)	0.0233 (8)	0.0034 (7)	0.0068 (6)	0.0055 (7)
C6	0.0227 (9)	0.0218 (9)	0.0243 (9)	0.0035 (7)	0.0045 (7)	0.0061 (7)
N7	0.0240 (8)	0.0257 (8)	0.0248 (8)	0.0056 (6)	0.0037 (6)	0.0052 (6)
C8	0.0208 (9)	0.0266 (9)	0.0229 (9)	0.0030 (7)	0.0069 (7)	0.0048 (7)
C9	0.0328 (10)	0.0299 (10)	0.0268 (10)	0.0058 (8)	−0.0002 (8)	0.0012 (8)
C10	0.0190 (8)	0.0221 (8)	0.0240 (8)	0.0030 (7)	0.0103 (7)	0.0056 (7)
C11	0.0197 (8)	0.0214 (8)	0.0229 (8)	0.0038 (7)	0.0089 (6)	0.0064 (7)
C12	0.0234 (9)	0.0180 (8)	0.0228 (8)	0.0045 (7)	0.0051 (7)	0.0031 (7)
C13	0.0248 (9)	0.0239 (9)	0.0328 (10)	0.0042 (7)	0.0091 (7)	0.0095 (8)
C14	0.0374 (11)	0.0252 (9)	0.0314 (10)	0.0062 (8)	0.0088 (8)	0.0121 (8)
C15	0.0362 (11)	0.0261 (9)	0.0293 (10)	0.0108 (8)	0.0001 (8)	0.0063 (8)
C16	0.0225 (9)	0.0315 (10)	0.0362 (11)	0.0067 (8)	0.0039 (8)	0.0053 (8)
C17	0.0247 (9)	0.0285 (10)	0.0291 (9)	0.0051 (7)	0.0088 (7)	0.0092 (8)
C18	0.0231 (9)	0.0224 (9)	0.0229 (8)	0.0018 (7)	0.0065 (7)	0.0036 (7)
O19	0.0385 (8)	0.0268 (7)	0.0525 (9)	−0.0009 (6)	0.0267 (7)	0.0083 (7)
C20	0.0237 (9)	0.0189 (8)	0.0227 (8)	0.0041 (7)	0.0053 (7)	0.0029 (7)
C21	0.0211 (8)	0.0188 (8)	0.0200 (8)	0.0041 (6)	0.0040 (6)	0.0019 (6)
C22	0.0189 (8)	0.0198 (8)	0.0184 (8)	0.0036 (6)	0.0027 (6)	0.0032 (6)
C23	0.0237 (9)	0.0167 (8)	0.0252 (9)	0.0050 (7)	0.0061 (7)	0.0041 (7)
C24	0.0235 (9)	0.0183 (8)	0.0249 (9)	0.0025 (7)	0.0076 (7)	0.0058 (7)
C51	0.0440 (13)	0.0422 (13)	0.0419 (13)	0.0048 (10)	0.0016 (10)	0.0096 (10)
C52	0.0454 (13)	0.0374 (12)	0.0354 (11)	0.0053 (10)	−0.0002 (9)	0.0090 (9)
C54	0.0376 (12)	0.0418 (13)	0.0482 (13)	0.0055 (10)	0.0113 (10)	0.0158 (11)
C55	0.0407 (13)	0.0438 (13)	0.0527 (14)	−0.0015 (10)	−0.0001 (11)	0.0131 (11)
O56	0.0483 (10)	0.0284 (8)	0.0802 (13)	0.0103 (7)	0.0120 (9)	0.0216 (8)

### Geometric parameters (Å, º)

**Table:**

O53—C54	1.318 (3)	C15—C16	1.384 (3)
O53—C52	1.496 (3)	C15—H15	0.95
Cl1—C1	1.7427 (18)	C16—C17	1.387 (3)
C1—C6	1.366 (3)	C16—H16	0.95
C1—C2	1.409 (3)	C17—H17	0.95
C2—C3	1.368 (3)	C18—O19	1.219 (2)
C2—H2	0.95	C18—C20	1.472 (2)
C3—C4	1.417 (3)	C20—C21	1.337 (2)
C3—H3	0.95	C20—H20	0.95
C4—N7	1.371 (2)	C21—C22	1.464 (2)
C4—C5	1.416 (3)	C21—H21	0.95
C5—C6	1.418 (2)	C22—C24^i^	1.399 (2)
C5—C11	1.424 (2)	C22—C23	1.402 (2)
C6—H6	0.95	C23—C24	1.385 (2)
N7—C8	1.319 (2)	C23—H23	0.95
C8—C10	1.431 (3)	C24—C22^i^	1.399 (2)
C8—C9	1.502 (3)	C24—H24	0.95
C9—H9A	0.98	C51—C52	1.507 (3)
C9—H9B	0.98	C51—H51A	0.98
C9—H9C	0.98	C51—H51B	0.98
C10—C11	1.376 (2)	C51—H51C	0.98
C10—C18	1.513 (2)	C52—H52A	0.99
C11—C12	1.493 (2)	C52—H52B	0.99
C12—C17	1.393 (3)	C54—O56	1.219 (3)
C12—C13	1.393 (3)	C54—C55	1.485 (3)
C13—C14	1.388 (3)	C55—H55A	0.98
C13—H13	0.95	C55—H55B	0.98
C14—C15	1.383 (3)	C55—H55C	0.98
C14—H14	0.95		
			
C54—O53—C52	115.38 (17)	C16—C15—H15	120
C6—C1—C2	122.05 (17)	C15—C16—C17	120.13 (18)
C6—C1—Cl1	118.43 (14)	C15—C16—H16	119.9
C2—C1—Cl1	119.52 (14)	C17—C16—H16	119.9
C3—C2—C1	118.92 (17)	C16—C17—C12	120.35 (17)
C3—C2—H2	120.5	C16—C17—H17	119.8
C1—C2—H2	120.5	C12—C17—H17	119.8
C2—C3—C4	121.30 (17)	O19—C18—C20	121.15 (16)
C2—C3—H3	119.4	O19—C18—C10	120.30 (15)
C4—C3—H3	119.4	C20—C18—C10	118.54 (15)
N7—C4—C5	122.46 (16)	C21—C20—C18	122.36 (16)
N7—C4—C3	118.70 (16)	C21—C20—H20	118.8
C5—C4—C3	118.84 (16)	C18—C20—H20	118.8
C4—C5—C6	119.40 (16)	C20—C21—C22	127.20 (16)
C4—C5—C11	118.38 (16)	C20—C21—H21	116.4
C6—C5—C11	122.22 (16)	C22—C21—H21	116.4
C1—C6—C5	119.46 (17)	C24^i^—C22—C23	118.45 (15)
C1—C6—H6	120.3	C24^i^—C22—C21	122.88 (16)
C5—C6—H6	120.3	C23—C22—C21	118.67 (15)
C8—N7—C4	118.54 (16)	C24—C23—C22	121.39 (16)
N7—C8—C10	122.57 (16)	C24—C23—H23	119.3
N7—C8—C9	117.80 (16)	C22—C23—H23	119.3
C10—C8—C9	119.64 (16)	C23—C24—C22^i^	120.16 (16)
C8—C9—H9A	109.5	C23—C24—H24	119.9
C8—C9—H9B	109.5	C22^i^—C24—H24	119.9
H9A—C9—H9B	109.5	C52—C51—H51A	109.5
C8—C9—H9C	109.5	C52—C51—H51B	109.5
H9A—C9—H9C	109.5	H51A—C51—H51B	109.5
H9B—C9—H9C	109.5	C52—C51—H51C	109.5
C11—C10—C8	120.02 (16)	H51A—C51—H51C	109.5
C11—C10—C18	119.87 (16)	H51B—C51—H51C	109.5
C8—C10—C18	120.11 (16)	O53—C52—C51	109.09 (18)
C10—C11—C5	118.01 (16)	O53—C52—H52A	109.9
C10—C11—C12	121.72 (16)	C51—C52—H52A	109.9
C5—C11—C12	120.27 (16)	O53—C52—H52B	109.9
C17—C12—C13	119.09 (17)	C51—C52—H52B	109.9
C17—C12—C11	120.53 (16)	H52A—C52—H52B	108.3
C13—C12—C11	120.37 (16)	O56—C54—O53	122.8 (2)
C14—C13—C12	120.34 (17)	O56—C54—C55	126.9 (2)
C14—C13—H13	119.8	O53—C54—C55	110.3 (2)
C12—C13—H13	119.8	C54—C55—H55A	109.5
C15—C14—C13	120.11 (18)	C54—C55—H55B	109.5
C15—C14—H14	119.9	H55A—C55—H55B	109.5
C13—C14—H14	119.9	C54—C55—H55C	109.5
C14—C15—C16	119.97 (18)	H55A—C55—H55C	109.5
C14—C15—H15	120	H55B—C55—H55C	109.5
			
C6—C1—C2—C3	1.2 (3)	C6—C5—C11—C12	−1.6 (2)
Cl1—C1—C2—C3	−178.76 (14)	C10—C11—C12—C17	103.4 (2)
C1—C2—C3—C4	0.2 (3)	C5—C11—C12—C17	−77.3 (2)
C2—C3—C4—N7	177.88 (17)	C10—C11—C12—C13	−77.7 (2)
C2—C3—C4—C5	−1.6 (3)	C5—C11—C12—C13	101.7 (2)
N7—C4—C5—C6	−177.88 (16)	C17—C12—C13—C14	−0.2 (3)
C3—C4—C5—C6	1.5 (2)	C11—C12—C13—C14	−179.20 (17)
N7—C4—C5—C11	1.4 (3)	C12—C13—C14—C15	−0.8 (3)
C3—C4—C5—C11	−179.16 (16)	C13—C14—C15—C16	0.8 (3)
C2—C1—C6—C5	−1.2 (3)	C14—C15—C16—C17	0.3 (3)
Cl1—C1—C6—C5	178.76 (13)	C15—C16—C17—C12	−1.3 (3)
C4—C5—C6—C1	−0.2 (3)	C13—C12—C17—C16	1.3 (3)
C11—C5—C6—C1	−179.47 (16)	C11—C12—C17—C16	−179.74 (17)
C5—C4—N7—C8	−0.2 (3)	C11—C10—C18—O19	92.4 (2)
C3—C4—N7—C8	−179.66 (16)	C8—C10—C18—O19	−87.7 (2)
C4—N7—C8—C10	−0.8 (3)	C11—C10—C18—C20	−88.2 (2)
C4—N7—C8—C9	179.53 (16)	C8—C10—C18—C20	91.7 (2)
N7—C8—C10—C11	0.7 (3)	O19—C18—C20—C21	172.69 (18)
C9—C8—C10—C11	−179.70 (16)	C10—C18—C20—C21	−6.7 (3)
N7—C8—C10—C18	−179.25 (16)	C18—C20—C21—C22	−178.00 (16)
C9—C8—C10—C18	0.4 (2)	C20—C21—C22—C24^i^	−3.7 (3)
C8—C10—C11—C5	0.6 (2)	C20—C21—C22—C23	177.15 (17)
C18—C10—C11—C5	−179.55 (14)	C24^i^—C22—C23—C24	−0.2 (3)
C8—C10—C11—C12	179.94 (15)	C21—C22—C23—C24	179.00 (16)
C18—C10—C11—C12	−0.2 (2)	C22—C23—C24—C22^i^	0.2 (3)
C4—C5—C11—C10	−1.5 (2)	C54—O53—C52—C51	−86.6 (2)
C6—C5—C11—C10	177.76 (15)	C52—O53—C54—O56	−3.7 (3)
C4—C5—C11—C12	179.09 (15)	C52—O53—C54—C55	176.92 (18)

Symmetry code: (i) −*x*+1, −*y*, −*z*.

### Hydrogen-bond geometry (Å, º)

Cg1 is the centroid of the C12–C17 ring.

**Table:**

*D*—H···*A*	*D*—H	H···*A*	*D*···*A*	*D*—H···*A*
C13—H13···O19^ii^	0.95	2.54	3.218 (2)	128
C23—H23···O53	0.95	2.57	3.468 (2)	157
C24—H24···O56^iii^	0.95	2.48	3.410 (3)	165
C51—H51*B*···*Cg*1^iv^	0.98	2.76	3.627 (3)	147

Symmetry codes: (ii) −*x*, −*y*, −*z*+1; (iii) −*x*+1, −*y*+1, −*z*; (iv) −*x*+1, −*y*+1, −*z*+1.
